# Novel adjustable traction “noose knot” method for colorectal endoscopic submucosal dissection

**DOI:** 10.1055/a-2227-6330

**Published:** 2024-01-23

**Authors:** Junki Tokura, Daisuke Ide, Keigo Suzuki, Chihiro Yasue, Akiko Chino, Masahiro Igarashi, Shoichi Saito

**Affiliations:** 1 Department of Gastroenterology, Cancer Institute Hospital, Japanese Foundation for Cancer Research, Tokyo, Japan

Traction techniques effectively aid in endoscopic submucosal dissection (ESD) by maintaining satisfactory traction during dissection 1 . We previously reported the usefulness of the pocket creation method using a traction device (PCM with TD) for colorectal ESD 2 3 . PCM with TD achieves stable en bloc resection and R0 dissection rates without adverse events. However, a single device may not provide sufficient traction, particularly in cases involving large lesions or a high degree of fibrosis. In these cases, additional traction is required, which increases the procedure difficulty. There are few previous reports on traction devices with adjustable traction force 4 . Herein, we present a novel traction device that enables adjustable traction through a method of ligating nylon threads, known as a “noose knot” ( Fig. 1 ).

**Fig. 1:**
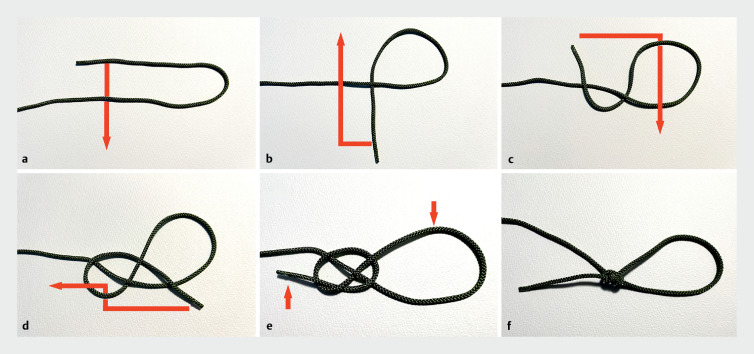
How to tie a “noose knot” of nylon thread. **a** Create a loop by passing the end of the thread through from the back down. **b** Move it from the front to the top left. **c** Pass it through the right loop from the back. **d** Pass it through the lower left loop from the back. **e** Finally, tighten the thread at the end and the top of the right loop. **f** Completed.

A 40-year-old woman presented with bloody stools and underwent lower gastrointestinal endoscopy, which revealed a 25-mm sessile serrated lesion in the ascending colon. Colorectal ESD was performed on the lesion using PCM with TD. Fig. 2 **a** and Fig. 2 **b** show the schemas of the PCM with TD 2 . Attaching the traction device to the anal side of the lesion and applying traction in the appropriate direction provides an adequate visual field, which enables the submucosal layer to be approached with ease. However, as in the present case, an adequate visual field may not be sustained during the procedure because of reduced traction ( Fig. 2 **c** ). Therefore, the nylon threads of the traction device were ligated as shown in Fig. 1 , allowing for increased traction by reducing the size of the ring during the procedure ( Fig. 2 **d,e** ). Consequently, the tumor was resected en bloc without complications ( Video 1 ). Pathological examination revealed a sessile-serrated lesion and confirmed complete resection of the tumor.

**Fig. 2:**
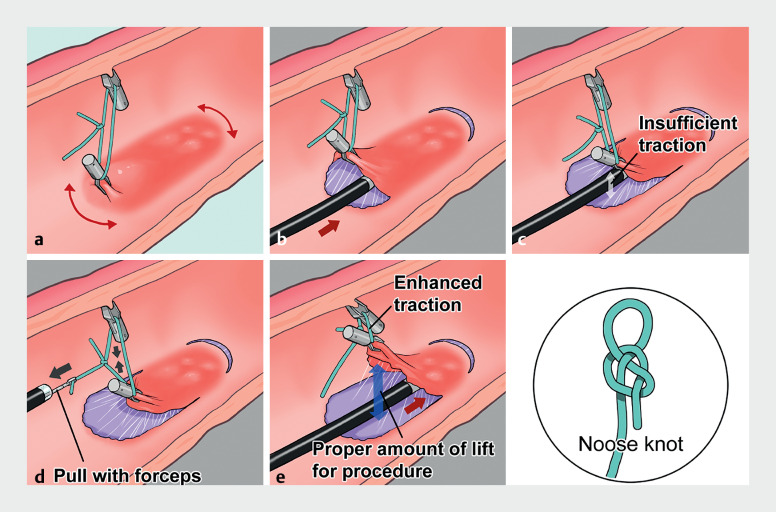
Schema of the pocket-creation method with a “noose knot” traction device. **a** The double arrow shows the incision line. The traction device stretches the mucosal and submucosal layers to facilitate creation of a mucosal flap and rapid formation of a submucosal pocket by incision. **b** After the mucosal pocket is created, an adequate visual field is obtained, which enables the submucosal layer to be approached with ease. **c** An adequate visual field may not be sustained during the procedure due to reduced traction. **d** Pulling the tip of the nylon thread of the “noose knot” traction device with forceps reduces the size of the ring. **e** More substantial traction is possible by reducing the size of the ring. Source: Medical Fig.

Preparation of the “noose knot” traction device for colorectal endoscopic submucosal dissection.Video 1

This “noose knot” traction device demonstrated an effective and concise method for enhancing traction. This method can be applied in any situation where traction is required during ESD of the gastrointestinal tract.

Endoscopy_UCTN_Code_TTT_1AQ_2AD

## Notes

Endoscopy E-Videos https://eref.thieme.de/e-videos
*E-Videos* is an open access online section of the journal *Endoscopy* , reporting on interesting cases and new techniques in gastroenterological endoscopy. All papers include a high-quality video and are published with a Creative Commons CC-BY license. Endoscopy E-Videos qualify for HINARI discounts and waivers and eligibility is automatically checked during the submission process. We grant 100% waivers to articles whose corresponding authors are based in Group A countries and 50% waivers to those who are based in Group B countries as classified by Research4Life (see: https://www.research4life.org/access/eligibility/ ). This section has its own submission website at https://mc.manuscriptcentral.com/e-videos .
